# Relationship between Resting-State Alpha Coherence and Cognitive Control in Individuals with Internet Gaming Disorder: A Multimodal Approach Based on Resting-State Electroencephalography and Event-Related Potentials

**DOI:** 10.3390/brainsci11121635

**Published:** 2021-12-11

**Authors:** Minkyung Park, So Young Yoo, Ji-Yoon Lee, Ja Wook Koo, Ung Gu Kang, Jung-Seok Choi

**Affiliations:** 1 Samsung Medical Center, Department of Psychiatry, Seoul 06351, Korea; reneedrv@gmail.com (M.P.); idiyuni91@gmail.com (J.-Y.L.); 2 SMG-SNU Boramae Medical Center, Department of Psychiatry, Seoul 07061, Korea; syyoomd@daum.net; 3 Emotion, Cognition and Behavior Research Group, Korea Brain Research Institute, Daegu 41062, Korea; jawook.koo@kbri.re.kr; 4 Department of Brain and Cognitive Sciences, Daegu Gyeongbuk Institute of Science and Technology, Daegu 42988, Korea; 5 Medical Research Center, Institute of Human Behavioral Medicine, Seoul National University, Seoul 03080, Korea; 6 Department of Psychiatry and Behavioral Science, College of Medicine, Seoul National University, Seoul 03080, Korea

**Keywords:** alpha coherence, Internet gaming disorder, inhibition, event-related potentials, Go/Nogo task

## Abstract

The human brain is constantly active, even at rest. Alpha coherence is an electroencephalography (EEG) rhythm that regulates functional connectivity between different brain regions. However, the relationships between resting-state alpha coherence and N2/P3 components associated with response inhibition and cognitive processes have not been investigated in addictive disorders. The present study investigated the relationships between alpha coherence during the resting state and N2/P3 components of event-related potentials during the Go/Nogo task in healthy controls (HCs) and patients with Internet gaming disorder (IGD). A total of 64 young adults (HC: *n* = 31; IGD: *n* = 33) participated in this study. Alpha coherence values at left fronto-central and bilateral centro-temporal electrode sites were significantly correlated with P3 latency in HCs, whereas inverse correlations were observed in patients with IGD. Furthermore, significant differences were observed in the correlation values between the groups. Our results suggest that patients with IGD lack dynamic interactions of functional connectivity between the fronto-centro-temporal regions during the resting state and the event-related potential (ERP) index during cognitive tasks. The findings of this study may have important implications for understanding the neurophysiological mechanisms linking resting-state EEG and task-related ERPs underlying IGD.

## 1. Introduction

In January 2020, the World Health Organization (WHO) declared the outbreak of the novel coronavirus (COVID-19) a global health emergency. COVID-19 has had a devastating impact on the lives of many and has resulted in physical and mental health crises [1,2]. The extension of stay-at-home measures and social distancing may be associated with heightened stress, as well as negative emotions such as anxiety and depression [3]. Several studies reported an increased consumption of alcohol and Internet gaming behavior during the pandemic [4]. Addictive behaviors such as Internet gaming and Internet use are considered to be putative coping strategies to deal with the negative consequences of social isolation and loneliness [5,6]. Internet gaming disorder (IGD) is characterized by the persistent and recurrent use of Internet gaming despite negative consequences [7]. The American Psychiatric Association included IGD in Section III of the Diagnostic and Statistical Manual of Mental Disorders, Fifth Edition (DSM-5) [8]. Additionally, the WHO released the 11th revision of the International Classification of Diseases (ICD-11), which include gaming disorder (GD) [9]. In the midst of the current global pandemic, the importance of mental health care cannot be overstated.

Over the last four decades, numerous studies have employed electroencephalography (EEG) to investigate the characteristics of addictive disorders. Resting-state EEG does not require active tasks, and EEG coherence is a useful tool for the diagnosis and treatment of diseases because it provides information on functional brain connectivity based on the coupling between two neuronal populations. EEG coherence analysis is a linear measure of the pairwise correlations of power spectra in the time domain between two electrode signals [10]. In this regard, a higher EEG coherence value indicates higher synchronization or intensive communication between neuronal populations [11]. A study reported that alpha coherence in the right hemisphere was higher in IGD patients with low resilience than in IGD patients with high resilience and healthy controls (HCs) [12]. A study on adolescents reported that compared to patients with attention deficit hyperactivity disorder (ADHD) only and HCs, patients with co-morbid ADHD and IGD had higher intra-hemispheric coherence in right parieto-occipital electrode sites at delta, theta, alpha, and beta frequencies [13]. Event-related potentials (ERPs) provide a wealth of information on brain function and have been harnessed extensively in the fields of neuroscience and psychiatry. Go/Nogo tasks have been employed to investigate the neurobiological mechanisms underlying inhibitory control and various cognitive functions [14,15]. A study using the Go/Nogo task reported that IGD patients exhibited delayed Nogo-N2 latency in the central area compared to HCs, which may reflect impaired response inhibition in IGD. Delayed latency has also been associated with IGD severity scores and impulsivity [16]. A source localization study conducted using the Go/Nogo task reported that patients with IGD exhibited reduced neuronal activity in the anterior cingulate cortex (ACC) and dysfunctional error-processing, entailing poor inhibition and decision-making [17].

The brain transmits and integrates multimodal information from internal and external environments via the efficient and flexible communication of neural networks. Cortical processing is known to be modulated by alpha coherence [18]. The brain is constantly active, and EEG alpha rhythms persist even during sleep or when not engaged in tasks [19]. In this regard, the default mode network (DMN) is defined as a set of brain regions that are more active at rest based on positron emission tomography (PET) and functional magnetic resonance imaging (fMRI) studies [20,21]. The DMN is a resting-state network (RSN) that plays a critical role in the integration of cognitive processes [22,23]. Synchronized activity of EEG alpha rhythms and RSNs across brain regions is a pattern of functional connectivity that is ubiquitously observed in healthy individuals during the resting state. Notably, the co-activation maps of EEG alpha rhythms are similar to those of RSNs at rest [24,25]. Furthermore, imaging studies have revealed decreased functional connectivity of RSNs when individuals are engaged in higher cognitive tasks, such as working memory tasks [26]. Various fMRI studies have also reported altered functional connectivity of RSNs in IGD patients during both resting state and active tasks [27,28].

Although numerous EEG-based studies have reported that neurophysiological alterations in brain function are associated with IGD, the association between EEG alpha coherence and ERP components in relation to IGD pathophysiology remains unclear. In this regard, it is important to elucidate the mechanisms of cortical neuronal synchronization underlying both resting-state brain function and the performance of cognitive tasks. Thus, this study aimed to explore the neurophysiological mechanisms of IGD via combined alpha coherence in resting-state EEG and ERP components. Based on previous studies, we hypothesized that the relationship between EEG alpha coherence at rest and Go/Nogo ERP components in patients with IGD would differ to that in HCs, i.e., increased alpha coherence in patients with IGD would be associated with ERP changes, indicating altered response inhibition.

## 2. Materials and Methods

### 2.1. Participants and Clinical Assessments

Participants between 18 and 39 years of age were recruited from the SMG-SNU Boramae Medical Center and via an Internet advertisement. Of 69 participants, five were excluded due to severe motion-related artifacts. In total, 64 young adults were included in the study. Of patients, 33 (31 men and 2 women) were classified as having IGD diagnosed by a psychiatrist and were assessed for comorbid disorders based on a clinician-administered interview and the criteria of the DSM-5 [8,29]. All subjects with IGD spent more than 4 h per day and/or 30 h per week playing Internet games. Thirty-one healthy individuals (26 men and 5 women) who played Internet games for less than 2 h per day participated in this study. All participants were interviewed using the Mini-International Neuropsychiatric Interview to assess past and current psychiatric diagnoses [30]. Disorder severity was evaluated using the Internet Addiction Test (IAT) [31]. Intelligence quotient (IQ) was estimated using the Wechsler Adult Intelligence Scale IV [32]. Exclusion criteria were a history of intellectual disability, significant head injury, psychotic disorder, and substance use disorder (with the exception of nicotine). All participants were right-handed and were medication-naïve at the time of assessment. Anxiety and depressive symptoms were assessed using the Beck’s Anxiety Inventory (BAI) [33] and Beck’s Depression Inventory (BDI) [34].

### 2.2. EEG Acquisition and Analysis

**Figure 1:**
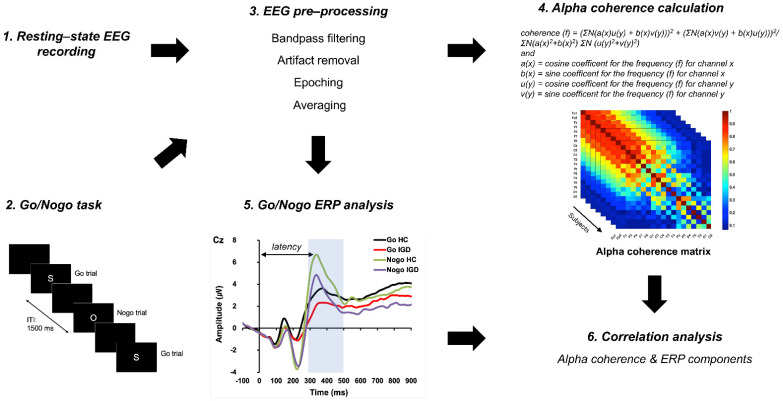
Schematic flow of this study.

Participants were seated in a comfortable chair in a dimly lit and electrically shielded room. Participants were instructed to stay relaxed and to avoid any body movements throughout the experiment. EEG data were recorded at a sampling rate of 1000 Hz with an online filter of 0.05–100 Hz using 64 Ag/AgCl electrodes based on the modified international 10–20 system (NeuroScan SynAmps2 (Compumedics USA, El Paso, TX, USA)). The electrodes at the linked mastoid sites served as reference electrodes, and the ground electrode was placed between the FPz and Fz electrode sites. Horizontal electrooculogram (EOG) signals were recorded with electrodes at the outer canthus of each eye, and vertical EOG signals were obtained from above and below the left. Gross artifacts, such as movement artifacts, were visually monitored and removed by an expert. Eye movements and eye blinks were removed using a regression procedure implemented in Curry 7 (Compumedics, Charlotte, NC) [35]. Epochs with EEG signals exceeding ±100 μV were excluded from further analysis. Electrode resistance was maintained below 5 kΩ. The schematic flow of this study is shown in Figure 1.

### 2.3. Resting-State Alpha Coherence

Resting-state EEG data were recorded for 5 min with eyes closed. Resting-state EEG activity was analyzed using Neuroguide Deluxe 2.6.1 software (Applied Neuroscience, Inc.; St. Petersburg, FL, USA). Linked ears reference data were used for further coherence analysis in offline analysis [36]. The following 19 channels were selected to generate resting-state EEG coherence values: FP1, FP2, F7, F3, Fz, F4, F8, T3, C3, Cz, C4, T4, T5, P3, Pz, P4, T6, O1, and O2. Artifact-free 2-sec epochs of EEG activity were converted into the frequency domain using the fast Fourier transformation and averaged over the alpha (8–12 Hz) frequency band. Alpha coherence was calculated using the following equation [37]:

Coherence (f) = (ΣN(a(x)u(y) + b(x)v(y)))2 + (ΣN(a(x)v(y) + b(x)u(y)))2/ΣN(a(x)2 + b(x)2) ΣN (u(y)2 + v(y)2)

whereby

a(x) = cosine coefficent for the frequency (f) for channel x

b(x) = sine coefficent for the frequency (f) for channel x

u(y) = cosine coefficent for the frequency (f) for channel y

v(y) = sine coefficent for the frequency (f) for channel y

Thus, 171 alpha coherence values were extracted. Four inter-hemispheric coherence values were evaluated between the frontal (F3–F4), central (C3–C4), temporal (T3–T4), and parietal (P3–P4) pairs of electrodes. Intra-hemispheric coherence values were examined using the following 12 electrode pairs: F3–C3, F3–T3, F3–P3, C3–T3, C3–P3, T3–P3, F4–C4, F4–T4, F4–P4, C4–T4, C4–P4, and T4–P4.

### 2.4. Go/Nogo Task

Go/Nogo task stimuli, which consisted of the letters “S” and “O” printed in white, were pseudorandomly generated using STIM 2. Participants were instructed to respond by pressing a button as accurately and quickly as possible to frequent Go stimuli (“S”, 70.7% probability) and to withhold responses to infrequent NoGo stimuli (“O”, 29.3% probability). The duration of each stimulus was 300 ms, and the intertrial interval (ITI) was 1500 ms. The experiment consisted of three blocks, with 200 trials in each block. The recorded electrophysiological data were processed offline using Curry 7 to extract ERP features. The data were recomputed using the average reference. The data were passed through a 0.1–30 Hz bandpass filter and segmented in epochs from 100 ms prior to the onset of stimuli to 900 ms post-stimulus. The baseline was corrected using the averaged pre-stimulus voltage. For the Go/Nogo task, N2 and P3 amplitudes and latencies for each condition were analyzed. Go-N2 and Nogo-N2 were identified as the largest negative peaks of the grand-averaged waveforms at 150–300 ms post-stimulus onset at the midline fronto-central (Fz and FCz) electrode sites. Go-P3 and NoGo-P3 were defined as the most positive deflections in the latency range of 250–500 ms at the midline centro-parietal (Cz, CPz, and Pz) electrode sites.

### 2.5. Statistical Analysis

R version 4.0.2 (R Development Core Team, Vienna, Austria) in RStudio (RStudio Team, 2020) was used for correlation analyses. The relationships among alpha coherence variables and ERPs were analyzed using Pearson’s correlation analysis. The Fisher-z method was used to assess the differences between two correlations. Statistical analyses were performed using SPSS software (ver. 22.0; IBM Corp., Armonk, NY, USA) for demographic, clinical, alpha coherence, and ERP analyses. All statistical analyses were two-tailed, with the significance level set at 0.05. Demographic and clinical characteristics were compared between groups using t-tests and χ² tests. Analysis of covariance with IQ as a covariate was performed to analyze group differences in behavioral data. For the alpha coherence analysis, intrahemispheric and interhemispheric coherence values were analyzed separately. Repeated-measures analysis of variance (ANOVA) with IQ as a covariate was performed on intrahemispheric coherence data, with region (fronto-central, fronto-temporal, fronto-parietal, centro-temporal, centro-parietal, and temporo-parietal) and hemisphere (left and right) as the within-subject factors and group as the between-subject factor. Repeated measures ANOVA with IQ as a covariate was performed on interhemispheric coherence data, with region (F3–F4, C3–C4, T3–T4, and P3–P4) as the within-subject factor and group as the between-subject factor. A cortical map of alpha coherence was visualized using the BrainNet Viewer for MATLAB R2017b [38].

For the Go/Nogo ERP analysis, the amplitudes and latencies of the N2 and P3 components were analyzed separately. Repeated measures ANOVA with IQ as a covariate was performed on Go-Nogo ERP data, with electrode site (Fz and FCz for N2; Cz, CPz, and Pz for P3) as the within-subject factor and group as the between-subject factor. Where necessary, Greenhouse-Geisser corrections were applied to correct for potential lack of sphericity. The Bonferroni method was used for post-hoc analysis.

### 2.6. Research Ethics

The study procedures were conducted in accordance with the Declaration of Helsinki and were approved by the Institutional Review Board of (blinded for review). All participants received detailed information regarding the study aims and procedures.

## 3. Results

### 3.1. Demographic Characteristics

The demographic and clinical characteristics of the participants are summarized in Table 1. No between-group differences in sex or age were observed. Significant group differences were observed in education, IQ, Y-IAT, BDI, and BAI scores. Compared with HCs, patients with IGD reported lower education years (t(62) = 2.859, *p* < 0.01) and IQ (t(62) = 4.639, *p* < 0.001) and higher Y-IAT (t(62) = −8.453, *p* < 0.001), BDI (t(62) = −7.354, *p* < 0.001), and BAI scores (t(62) = −5.020, *p* < 0.001).

### 3.2. Behavioral Results

In the Go condition, no significant between-group differences were observed in correct Go hit reaction times (RTs) or accuracy. In the Nogo condition, no significant between-group differences were observed in Nogo false alarm RTs or incorrect rates (see Table 2).

### 3.3. Correlations between Alpha Coherence and ERPs

**Figure 2:**
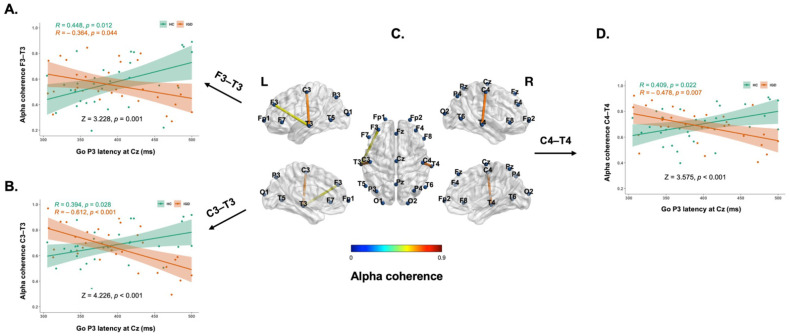
Inverse patterns of correlations between alpha coherence in the left fronto-temporal (F3–T3) (**A**), centro-temporal (C3–T3) (**B**) and right centro-temporal (C4–T4) (**D**) areas and the latency of Go-P3 in the healthy control (HC) and Internet gaming disorder (IGD) groups. HC people had positive correlation between alpha coherence and the latency of Go-P3 elicited with the Go/Nogo task, whereas IGD patients had negative correlation between alpha coherence and the latency of P3. (**C**) Cortical representation of the alpha coherence. The lines represent alpha coherence between electrode regions. (Left) Lateral and medial views of left hemisphere for fronto-temporal and centro-temporal alpha coherence. (Center) Dorsal view of inter and intra-hemispheric alpha coherence. (Right) Lateral and medial views of right hemisphere cento-temporal alpha coherence.

Pearson’s correlation analysis was performed for alpha coherence of resting EEG and N2/P3 components of the Go/Nogo task. In the present study, only statistically significant differences between groups and significant correlation results are reported. Significant positive relationships between Go-P3 latency at Cz and F3–T3 (r = 0.448, *p* = 0.012), F4–T4 (r = 0.374, *p* = 0.038), C3–T3 (r = 0.394, *p* = 0.028), C4–T4 (r = 0.409, *p* = 0.022), and T3–T4 (r = 0.418, *p* = 0.019) alpha coherence were identified in the HC group. In contrast, significant negative relationships between Go-P3 latency at Cz and F3–T3 (r = –0.364, *p* = 0.044), C3–T3 (r = –0.612, *p* < 0.001), T3–P3 (r = –0.422, *p* = 0.018), and C4–T4 (r = –0.478, *p* = 0.007) alpha coherence were observed in the IGD group. Group differences were observed in the relationship between Go-P3 latency at Cz and F3–T3 (Z = 3.228, *p* = 0.001), C3–T3 (Z = 4.226, *p* < 0.001), and C4–T4 (Z = 3.575, *p* < 0.001) alpha coherence. The correlations between alpha coherence and P3 latency are presented in Figure 2.

No significant correlations were observed between alpha coherence and BDI, BAI, and IAT scores, or between Go-P3 latency at Cz and BAI and IAT scores in patients with IGD. Significant positive correlations were observed between Go-P3 latency at Cz and BDI scores (r = 0.409, *p* = 0.022) and between the latency of Go-P3 and RT (r = 0.617, *p* < 0.001) in the IGD group.

### 3.4. Between-Group Differences in Alpha Coherence

No significant group difference (F(1, 61) = 0.849, *p* = 0.360, *η_p_*^2^ = 0.014) or significant group × region interaction (F(2.613, 61) = 0.461, *p* = 0.683, *η_p_*^2^ = 0.008) was observed for interhemispheric alpha coherence. No significant effect of group (F(1, 61) = 0.44, *p* = 0.834, *η_p_*^2^ = 0.001) or significant interactions of region × group (F(2.683, 61) = 0.243, *p* = 0.845, *η_p_*^2^ = 0.004), hemi × group (F(1, 61) = 0.008, *p* = 0.931, *η_p_*^2^ < 0.001), region × hemi (F(3.439, 61) = 0.676, *p* = 0.587, *η_p_*^2^ = 0.011), or region × hemi × group (F(3.439, 61) = 0.366, *p* = 0.804, *η_p_*^2^ = 0.006) were observed for interhemispheric alpha coherence.

### 3.5. Between-Group Differences in ERP Results

There was a significant main effect of inhibitory condition (Go/NoGo) on N2 amplitude (F(1, 57) = 6.781, *p* = 0.012, *η_p_*^2^ = 0.106). No significant group differences or interaction effects were observed for N2 amplitude and latency or P3 amplitude and latency.

## 4. Discussion

In this study, we investigated group differences in the correlations between resting-state EEG alpha coherence and Go/Nogo ERP components in participants with IGD and HCs. Notably, we identified significant group differences in the correlations between Go-P3 latency and alpha coherence of the left fronto-temporal and bilateral centro-temporal regions. In the HC group, positive correlations were observed between alpha coherence in the left fronto-temporal and bilateral centro-temporal areas and Go-P3 latency. Go-P3 reflects the subsequent processes of response inhibition related to stimulus evaluation or allocation of attentional resources [39]. P3 latency is considered an index of neural speed or brain efficiency [40,41]. In this regard, it is conceivable that HCs with high alpha coherence tended to adopt more cautious decision-making strategies, as reflected by the prolongation of P3, compared to HCs with low alpha coherence.

The relationship between intrahemispheric EEG alpha coherence and Go-P3 latency was reversed in patients with IGD compared to that in HCs. Although no significant group differences were observed in the latency of P3 components, there is considerable evidence indicating deficits in response inhibition and executive function associated with P3 components in IGD. In addition, P3 latency was positively correlated with BDI scores in patients with IGD, which suggests that IGD patients with more severe depression had impaired response inhibition accompanied by delayed P3 latency. However, we did not observe significant group differences in resting-state intrahemispheric EEG alpha coherence. Furthermore, no significant correlation was observed between intrahemispheric alpha coherence values and IGD severity scores in patients with IGD. These results suggest that resting-state intrahemispheric EEG alpha coherence values alone cannot be considered as a state marker in patients with IGD.

Effective communication among brain regions is regulated by alpha rhythms and requires a functionally well-organized connection of neuronal activity across cortical regions. Although no significant group differences were observed for alpha coherence and ERP components, we identified significant group differences in the correlations between Go-P3 latency and intrahemispheric coherence in alpha rhythms. Our findings suggest that the information-processing regulatory system in patients with IGD was altered compared to that in HCs during the transition from resting state to task activity. Furthermore, compared to the HC group, patients with IGD exhibited poor performance on the Go/Nogo task, with a trend for lower accuracy and higher rate of incorrect responses, but this did not reach statistical significance. In both groups, the rate of incorrect responses was less than 15%, and response inhibition in the behavioral task was relatively good, which could underpin the relative ease of performing this task. These findings are consistent with the behavioral results of previous fMRI studies employing the Go/Nogo task [42,43].

The current findings are partly consistent with those of previous studies with respect to alpha coherence. One study reported that alpha coherence was higher in patients with low resilience than in patients with high resilience and HCs; this was associated with a higher prevalence of depression and stress in IGD patients with low resilience. In addition, the study reported that resilience and alpha coherence were positively correlated in the HC group and negatively correlated in patients with IGD [12]. The inverse relationships between resilience and alpha coherence are in accordance with the results of this study, which identified significant group differences in the correlations between Go-P3 latency and alpha rhythms. Another study reported that interhemispheric alpha coherence in the frontal regions was lower and intrahemispheric alpha coherence in the left parietal-occipital electrode pairs was higher in patients with comorbid IGD and major depressive disorder (MDD) than in patients with MDD without comorbidities, suggesting that patients with comorbid IGD and MDD could be vulnerable to attention problems [44].

Studies on alcohol use disorder (AUD) have identified altered functional connectivity between cortical regions in patients with AUD, and these alterations in EEG coherence may serve as an endophenotype for AUD. Studies have reported lower theta and alpha coherence in patients with AUD [45,46] and higher alpha coherence in the left temporo-occipital regions in patients with AUD [47].

An fMRI study using the Go/Nogo task reported that activation of the right supplementary motor area (SMA)/preSMA, a component of the frontostriatal network involved in processing response inhibition, was lower in patients with IGD [42]. Further, excessive Internet gaming behavior was associated with regulatory failure of the frontostriatal network [48]. Multimodal studies have demonstrated that activation of the RSN is associated with EEG alpha rhythms [49,50]. Neuroimaging studies have highlighted several similarities in clinical characteristics, including depression, anxiety, and alterations in cognitive function and inhibition between IGD and AUD [51,52]. However, alterations in alpha coherence were not observed in patients with IGD, unlike in patients with AUD. Based on previous studies, alpha coherence in patients with IGD may be affected by several clinical factors such as resilience and depression, which are underscored by distinct neurophysiological mechanisms to those underlying AUD.

Inconsistent with our hypothesis, the negative relationship between P3 and alpha coherence at fronto-central and centro-temporal electrode sites in patients with IGD differed from that in the HC group. This result suggests that IGD patients with lower alpha coherence may have poor P3 performance or delayed latency, given that weaker functional connectivity of alpha coherence is likely related to the delay in response inhibition. Given the paucity of studies on alpha coherence in IGD, we were unable to conclude from the literature whether the observed alpha coherence values were related to altered or normal mechanisms underscoring IGD. Further research on the relationship between alpha coherence and ERPs is warranted to improve our understanding of the underlying mechanisms and facilitate the development of treatments for IGD.

This study had several limitations that should be considered. First, we did not compare IGD with substance use disorder (SUD) such as AUD and other behavioral addictive disorders such as gambling disorder. Thus, the results of this study may not be generalizable to other addictive disorders. Second, we did not investigate electrophysiological indices of error processing such as error-related negativity (ERN) and error positivity (Pe) of response-locked ERP components elicited during a Go/Nogo task. Third, sensor-level EEG analysis has low spatial resolution and lacks information about the origin of brain activity. Hence, more studies are needed to examine EEG source localization and resting-state fMRI with high spatial resolution to elucidate the neurobiological features of IGD and facilitate the diagnosis and/or prevent the development of IGD. Despite these limitations, to our knowledge, this is the first study to investigate the association between resting-state EEG alpha coherence and ERP features in patients with IGD.

## 5. Conclusions

This study identified distinct patterns of associations between resting-state EEG alpha coherence and ERP components elicited with the Go/Nogo task in HCs and patients with IGD. Our results suggest that patients with IGD lack dynamic interactions of functional connectivity between fronto-centro-temporal regions during the resting state and the ERP index during cognitive tasks. The findings of this study provide novel insight into the neurophysiological mechanisms underlying IGD.

## Figures and Tables

**Table 1:** Demographic and clinical characteristics of study participants.

	Healthy Control (*n* = 31)	Internet Gaming Disorder (*n* = 33)	*X*^2^ or t	*p*
	Mean (SD)	Mean (SD)		
Sex (male/female)	26/5	31/2	1.663	0.197
Age (year)	25.00 (3.17)	25.00 (5.14)	0.000	1.000
Education (year)	14.71 (2.02)	13.33 (1.83)	2.859	0.006 **
IQ	117.19 (11.44)	102.79 (13.27)	4.639	<0.001 ***
Young’s Internet Addiction Test	33.00 (11.12)	60.82 (14.82)	−8.453	<0.001 ***
BDI	4.00 (4.62)	17.33 (9.04)	−7.354	<0.001 ***
BAI	3.53 (5.25)	14.92 (11.56)	−5.020	<0.001 ***

Note. ** = *p* < 0.01; *** = *p* < 0.001; SD = Standard deviation; IQ = intelligence quotient; BDI = Beck Depression Inventory; BAI = Beck Anxiety Inventory.

**Table 2:** This is a table. Behavioral data elicited with the Go/Nogo Task.

	Healthy Control (*n* = 31)	Internet Gaming Disorder (*n* = 33)	F	*p*
	Mean (SD)	Mean (SD)		
Accuracy (%)	98.93 (2.30)	96.91 (7.04)	0.016	0.901
Reaction time (ms)	264.41 (28.82)	263.68 (40.40)	0.009	0.925
Incorrect rate (%)	11.69 (8.20)	13.65 (11.32)	0.002	0.966
Nogo Reaction time (ms)	232.83 (40.45)	236.23 (48.73)	0.064	0.801

Note. SD = Standard deviation.

## Data Availability

The data presented in this study are available on reasonable request from the corresponding author.
